# Current decline in the number and size of *Galba truncatula* and *Omphiscola glabra* populations, intermediate hosts of *Fasciola hepatica*, on the acidic soils of Central France

**DOI:** 10.1051/parasite/2016055

**Published:** 2016-10-24

**Authors:** Gilles Dreyfuss, Philippe Vignoles, Daniel Rondelaud

**Affiliations:** 1 Laboratory of Parasitology, Faculty of Pharmacy 87025 Limoges Cedex France

**Keywords:** *Galba truncatula*, *Omphiscola glabra*, Population decline, Snail density, Snail habitat

## Abstract

Field investigations on the habitats colonized by *Galba truncatula* or *Omphiscola glabra* were carried out on 162 farms of the Limousin region, Central France, to determine whether there is currently a decline in the number and size of snail populations. Seven types of snail habitats were considered here. Compared to the numbers of snail populations recorded from 1976 to 1992, the values noted from 2013 to 2016 were significantly lower, with a decline rate of 34% for *G. truncatula* and 23% for *O. glabra*. Variations in this decline rate with the type of snail habitat were also noted. The greatest decreases in the numbers of snail populations were noted for spring heads located in meadows and for road ditches, while the lowest were noted for open drainage furrows present in meadows. The distribution of these habitats according to their area did not show any significant change over time. In contrast, overwintering snails were significantly less numerous in 2013–2016 in five types of habitats for *G. truncatula* and in three types only for *O. glabra.* Several causes underlie this population decline. Among them, the current development of mechanical cleaning in open drainage systems and road ditches, that of subsurface drainage in meadows, and regular gyro-crushing of vegetation around temporary spring heads were the most important.

## Abstract (fr)

Des investigations de terrain ont été réalisées sur les habitats colonisés par *Galba truncatula* ou *Omphiscola glabra* dans 162 fermes de la région Limousin (Centre de la France) afin de déterminer s’il existe un déclin actuel dans le nombre et la taille des populations. Sept types d’habitats ont été ici considérés. Par rapport aux nombres de populations décomptées de 1976 à 1992, les valeurs notées en 2013-2016 sont significativement plus faibles avec un déclin de 34 % pour *G. truncatula* et de 23 % pour *O. glabra*. Des variations ont été notées dans ces pourcentages par rapport au type d’habitat. Les plus fortes baisses dans le nombre des populations ont été notées dans le cas des sources dans les prairies et des fossés de route, alors que les plus faibles ont été relevées dans les rigoles de drainage superficiel dans les prairies. La distribution de ces habitats en fonction de leur superficie n’a pas présenté de changement significatif au cours du temps. Par contre, les mollusques transhivernants sont significativement moins nombreux dans cinq types d’habitats pour *G. truncatula* et dans trois types seulement pour *O. glabra*. Plusieurs causes sont à l’origine de ce déclin dans les populations. Parmi celles-ci, le développement actuel du curage mécanique dans les réseaux de drainage superficiel et les fossés de route, celui du drainage souterrain dans les prairies et le gyrobroyage régulier de la végétation autour des sources temporaires sont les plus importants.

## Introduction

*Galba truncatula* O.F. Müller, 1774 [14] and *Omphiscola glabra* O.F. Müller, 1774 [14] are freshwater pulmonate gastropods whose populations are still quite high in Western European countries. *G. truncatula* has a poor reputation [11, 26, 30], as it is known to be the common intermediate host of the parasite *Fasciola hepatica* Linnaeus, 1758 [9]. The second species was also reported by Abrous et al. [1, 2, 23] as an occasional host of the same digenean. These two snail species are frequently found on the cristallophyllian soils of the Limousin region (Central France). Among the 11,992 watered sites investigated by Vareille-Morel et al. [28] on 361 farms between 1976 and 1999, 7709 were colonized by *G. truncatula*: 60.5% of these snail habitats were found at the peripheral extremity of open drainage furrows in meadows, 15.3% around hillside spring heads, 12.3% in road ditches, 5.3% along the main drainage ditches, and the others along pond, stream, or river banks [28]. Contrary to *G. truncatula*, the habitats colonized by *O. glabra* were few in number (3137), even though they were found in the same types of watered sites. Among these habitats, 42.8% were found in open drainage furrows and/or temporary springs, 28.2% in road ditches, 13.3% along main drainage ditches, and the others along pond or stream banks [28]. Open-air cattle or sheep breeding in these permanent pastures throughout the year allows for infection with *F. hepatica*, with prevalence of natural infections that is still rather high in the Limousin region. In the French department of Corrèze, Mage et al. [10] have reported a mean prevalence of 17.1% in local cattle, with annual variations from 11.2% to 25.2% over a period of 10 years (1990–1999).

Since the 1980s, some habitats colonized by either lymnaeid have been lost in these permanent meadows because new agricultural methods are used. Among them, subsurface drainage and rush gyro-crushing around hillside springs have become common on most farms over the past 45 years [21]. At the present time, no study has been carried out in the Limousin region to evaluate the effect of these agricultural methods on the number and size of lymnaeid populations. According to Seddon et al. [24], *G. truncatula* is a widespread species and no specific threats exist at the global level, as this snail can be found in polluted waters and is known as a colonizing species in many temporary habitats. In contrast, there was generalized decline in the number and size of *O. glabra* populations throughout the geographical range of this species. The snail is currently listed as critically endangered in the Republic of Ireland, endangered in Germany, and vulnerable in Great Britain, the Netherlands, and Sweden [4, 16, 32, 33]. In view of this information, it was interesting to assess the decrease in the number of these lymnaeid populations and their size depending on their location in a breeding region and the type of snail habitat. A comparative study of the results from two series of investigations was thus performed. The first series was carried out from 1976 to 1992 on 162 farms located in the three departments of Limousin, i.e. Corrèze, Creuse, and Haute Vienne [22, 31]. The second series of investigations was performed from 2013 to 2016 on the same farms.

## Materials and methods

### Farms studied

The 361 farms investigated by Vareille-Morel et al. (2007) were located on cristallophyllian or metamorphic soils and bred cattle or sheep. Their altitude ranged from 190 to 500 m above sea level in 97.5% of cases. Permanent meadows present in these farms were hygro-mesophilous and were alternately subject to grazing and mowing. An open drainage system was generally dug in these grasslands. Owing to the nature of soils, the pH of running water ranged from 5.6 to 7 and the level of dissolved calcium was generally less than 20 mg/L [8]. All these farms have a continental climate, modulated by wet winds that come from the Atlantic Ocean. Depending on the year, the mean annual rainfall ranged from 800 to 1000 mm, while the mean annual temperature was 10°–10.5 °C on most farms [22].

**Figure 1.**
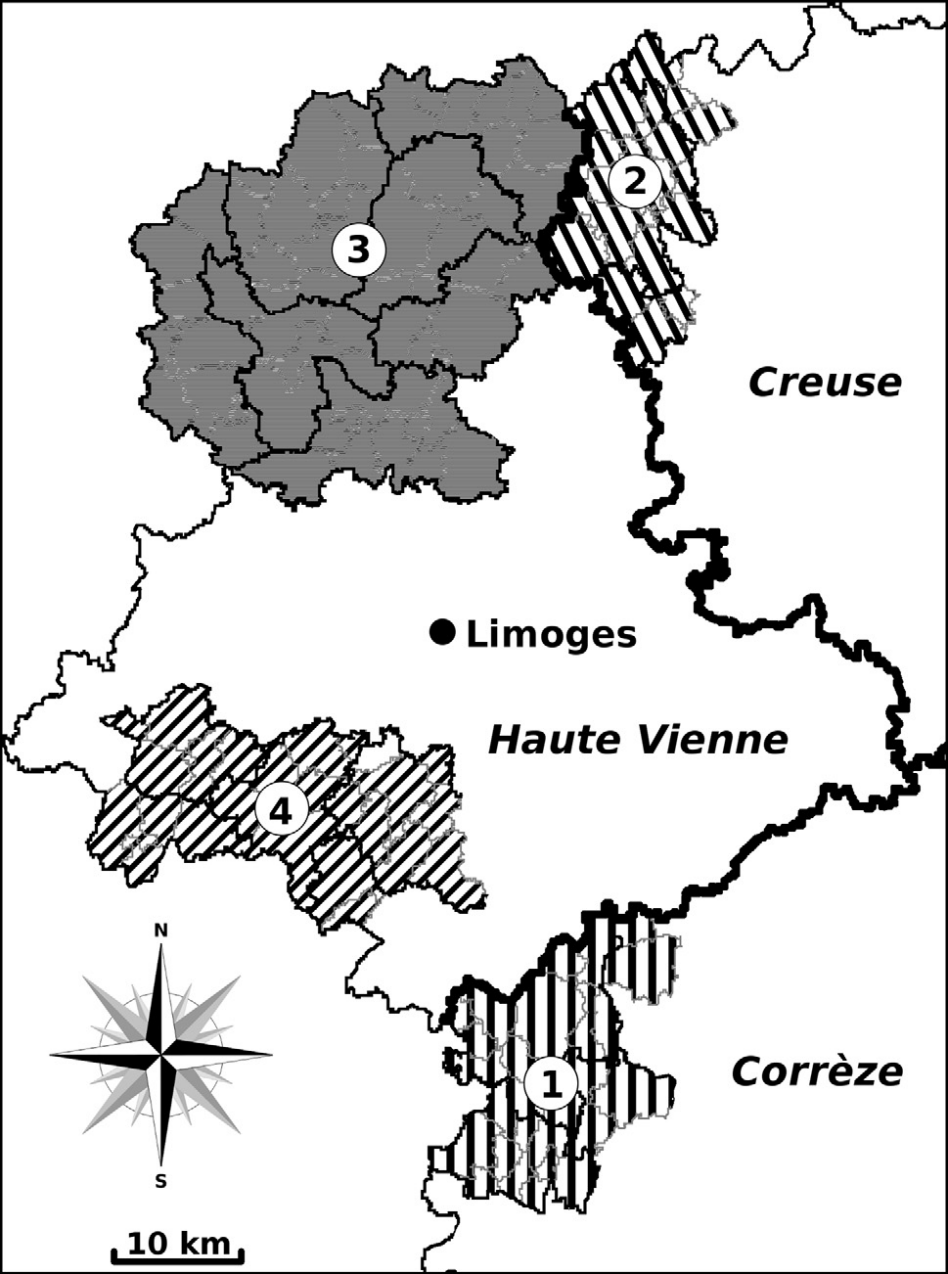
Map showing the geographical location of the 162 cattle- and sheep-breeding farms in the departments of Corrèze, Creuse, and Haute Vienne (Central France). Natural regions: 1, western plateaus of Corrèze; 2, north-western and western plateaus of Creuse; 3, northern third of Haute Vienne; and 4, south-west and south of the same department.

Among this range of farms, a total of 162 were selected according to the following three criteria: (i) these farms were located in a natural zone of the Limousin region, (ii) their activity concerning cattle or sheep breeding had not changed over at least the past 30 years, and (iii) their pastures were colonized by both lymnaeids. Four natural zones (Table 1 and Fig. 1) were selected: (i) the western plateaus of Corrèze (27 farms), (ii) the north-western and western plateaus of Creuse (22 farms), (iii) the northern third of Haute Vienne (77 farms), and (iv) the south-west and south of the same department (36 farms). In these natural zones, most farmers bred cattle (Corrèze, Creuse), sheep (northern Haute Vienne), and cattle and/or sheep (southern and south-western Haute Vienne).

**Table 1.** Number of cattle- and sheep-breeding farms investigated in the three departments of Limousin in relation to their location in a natural region and the type of breeding.

Natural region	Cantons*	Total number of farms	Number of farms	
			Cattle	Sheep
Western Corrèze	Allassac	27	9	2
	Uzerche		5	0
	L’Yssandonnais		10	1
North-western and western Creuse	Dun le Palestel	22	7	2
	Grand Bourg		6	2
	La Souterraine		4	1
Northern Haute Vienne	Bellac	77	7	35
	Châteauponsac		8	27
South and south-western Haute Vienne	Rochechouart	36	11	8
	St Yrieix la Perche		8	9
Totals	–	162	75	87

* French administrative subdivision grouping several municipalities.

From 1976 to 1992, a first series of investigations was carried out on these 162 farms by our team to detect snail habitats, identify lymnaeid species, and count overwintering snails. Other counts of habitats and overwintering snails were also performed from 2013 to 2016. These investigations were performed in March or April on the whole area of each farm because the habitats were waterlogged at that time and only contained snails of the overwintering generation.

### Protocol of investigations

Seven types of snail habitats were considered for either lymnaeid. The first four were located in meadows: (i) open drainage furrows, (ii) temporary or permanent spring heads on hillsides, (iii) open drainage ditches, and (iv) cattle-trampled areas. The other three types were (v) road or way ditches when they are waterlogged during winter and spring, (vi) small streams, and (vii) pools and ponds.

During the two series of investigations, the detection of habitats in each meadow and snail counts were performed by two persons over 30–40 min. In road ditches and along banks, the investigations were performed by a single person for 15–20 min per habitat. Depending on water levels, overwintering snails were counted by sight or after their collection using a sieve (mesh size, 3 mm). After each count, snails collected from their habitat were replaced into it. The area of each site was subsequently determined. Measurements of areas occupied by either lymnaeid were easy in the case of furrows, ditches, pools, ponds, and stream banks. Snail habitats located around spring heads and in trampled zones were drawn on maps and their area was determined in relation to their geometric form and dimensions.

On the 162 farms concerned by the second series of investigations, the herder was interviewed to determine the cause of population disappearance when the habitats of either species were no longer found.

### Parameters studied

These parameters were the number of snail habitats, the overall area of these sites, and the density of overwintering snails per m^2^ of habitat. To determine the distribution of *G. truncatula* habitats in relation to their area, values were given as follows: up to 1 m^2^, from 1.1 to 2 m^2^, from 2.1 to 3 m^2^, from 3.1 to 4 m^2^, and >4 m^2^. In the case of *O. glabra*, the areas were classified into the following four categories: up to 2 m^2^, from 2.1 to 5 m^2^, from 5.1 to 10 m^2^, and >10 m^2^. Similarly, the distribution of habitats in relation to snail densities was studied using four classes of densities for *G. truncatula* (≤10 snails/m^2^, from 10.1 to 25/m^2^, from 25.1 to 40/m^2^, and >40/m^2^) and four others for *O. glabra* (≤5 snails/m^2^, from 5.1 to 10/m^2^, from 10.1 to 15/m^2^, and >15/m^2^). These area and density classes were determined during the first series of investigations [22, 31]. This distribution of habitats into classes according to their areas and snail densities was preferred here to calculation of mean areas and mean densities, because these classes enabled us to limit the influence of extreme dispersions and to more easily detect any changes between the two periods of investigations.

The numbers of snail habitats detected on the 162 farms before 1993 and from 2013 to 2016 were compared using the unilateral Wilcoxon test on matched data (the same farms). The distributions of snail habitats based on their areas were compared using Fisher’s exact test. A similar protocol was applied for the distribution of habitats based on the density of overwintering snails. All these analyses were performed using R ×64 3.3.0 software [17].

## Results

### Number of snail populations

Table 2 lists the numbers of populations recorded before 1993 and from 2013 to 2016 in the seven types of habitats. In the case of *G. truncatula*, there was an overall decrease (a mean of 34%) in the number of these populations over time. However, this decline showed variations in relation to the type of snail habitat. The highest percentages were noted in the case of spring heads (65.3%), followed by road ditches (63.8%) and open drainage ditches (59.8%), by decreasing order. The lowest decrease in the number of these snail populations was noted in the case of open drainage furrows (21.3%). Similar findings were also noted for *O. glabra*, with an overall decline of 23.4% over time. The highest decreases in the numbers of *O. glabra* populations were noted for trampled areas (77.7%), followed by spring heads (70.9%) and road ditches (54.4%). The lowest decrease was found for snail habitats located in open drainage furrows (7.3%). These decreases between both periods of snail investigations were significant (*G. truncatula*: *W* = 36, *p* < 0.01; *O. glabra*: *W* = 36, *p* < 0.01) for each type of habitat.

**Table 2.** Number of snail habitats colonized by *Galba truncatula* and *Omphiscola glabra* on the 162 farms before 1993 and in 2013–2016 in relation to their type.

Type of snail habitat	Number of snail habitats (decline rate in %)			
	*Galba truncatula*		*Omphiscola glabra*	
	Before 1993	From 2013 to 2016	Before 1993	From 2013 to 2016
Drainage furrows	2032	1598 (21.3)	707	655 (7.3)
Road ditches	407	147 (63.8)	169	77 (54.4)
Spring heads	294	102 (65.3)	117	34 (70.9)
Drainage ditches	102	43 (57.9)	56	47 (16.0)
Streams	92	47 (48.9)	41	28 (31.7)
Pools and ponds	71	41 (42.2)	32	23 (28.1)
Trampled areas	17	11 (35.2)	9	2 (77.7)
All types	3015	1987 (34.0)	1131	866 (23.4)

No significant difference between the numbers of populations recorded before 1993 and in 2013–2016 was noted when the geographical location of farms in a natural region or the type of ruminant was considered.

### Area of snail habitats

Table 3 shows the distribution of *G. truncatula* populations in relation to the area of their habitat. In the 1987 habitats investigated before 1993, 41.1% had an area ranging from 1.1 to 2 m^2^. Values ranging from 2.1 to 3 m^2^ and >3 m^2^ were noted in 28% and 12.5% of these sites, respectively, while the area was ≤1 m^2^ in the other 18.2%. In the same habitats measured between 2013 and 2016, the area was ≤1 m^2^ in 18% of these sites, between 1.1 and 2 m^2^ in 42.2%, between 2.1 and 3 m^2^ in 25.2%, and higher than 3 m^2^ in the other 14.3%. No significant difference between the distributions of these habitats before 1993 and in 2013–2016 was noted, whatever the type of habitat.

**Table 3.** Distribution of 1987 habitats colonized by *Galba truncatula* on the 162 farms before 1993 and in 2013–2016 in relation to the type and area of habitat. The total number of snail habitats is given after each type of habitat (first column).

Parameters	Number of habitats colonized by *Galba truncatula*									
	Before 1993					From 2013 to 2016				
Snail area (m^2^)	0.1–1	1.1–2	2.1–3	3.1–4	>4	0.1–1	1.1–2	2.1–3	3.1–4	>4
Type of snail habitat										
Drainage furrows (1598)	314	734	463	85	2	306	752	413	127	0
Road ditches (147)	0	1	34	71	41	0	5	31	74	37
Spring heads (102)	41	54	7	0	0	45	55	2	0	0
Streams (47)	2	11	20	9	5	4	9	22	10	2
Drainage ditches (41)	6	17	13	5	0	4	18	15	3	1
Pools and ponds (41)	0	0	17	15	9	0	1	16	17	7
Trampled areas (11)	0	0	3	3	5	0	0	2	5	4
All types (1987)	363	817	557	188	62	359	840	501	236	51
Frequency (%)	18.2	41.1	28.0	9.4	3.1	18.0	42.2	25.2	11.8	2.5

In the *O. glabra* habitats investigated before 1993 (Table 4), 47.3% of habitats had areas ranging from 5.1 to 10 m^2^. Lower percentages were noted for the 0.1–2 m^2^ (13.7% of habitats) and 2.1–5 m^2^ (26.5%) areas, while the others had values greater than 10 m^2^ (12.3%). In 2013–2016, several slight changes were noted in the distribution of these habitats according to their area, but these changes were not significant, whatever the type of habitat.

**Table 4.** Distribution of 866 habitats colonized by *Omphiscola glabra* on the 162 farms before 1993 and in 2013–2016 in relation to the type and area of habitat. The total number of snail habitats is given after each type of habitat (first column).

Parameters	Number of habitats colonized by *Omphiscola glabra*							
	Before 1993				From 2013 to 2016			
Snail area (m^2^)	0.1–2	2.1–5	5.1–10	>10	0.1–2	2.1–5	5.1–10	>10
Type of snail habitat								
Drainage furrows (655)	58	157	351	89	61	169	347	78
Road ditches (77)	15	33	22	7	16	35	19	7
Drainage ditches (47)	7	9	24	7	8	11	19	9
Spring heads (34)	14	11	6	3	15	9	7	3
Streams (28)	6	16	5	1	9	12	6	1
Pools and ponds (23)	17	4	2	0	16	4	3	0
Trampled areas (2)	2	0	0	0	2	0	0	0
All types (866)	119	230	410	107	127	240	401	98
Frequency (%)	13.7	26.5	47.3	12.3	14.6	27.7	46.3	11.3

### Number of snails per population

Compared to the distribution of *G. truncatula* populations recorded before 1993 (Table 5), there was a significant change (*p* < 0.001) in values noted from 2013 to 2016. The overall frequency of habitats with 1 to 10 snails/m^2^ had increased from 20.4% (before 1995) to 42.7% (in 2013–2016). In contrast, the habitats containing 26–40 *G. truncatula*/m^2^ or a higher density were less numerous in 2013–2016 (11.5% and 0.8%, respectively, instead of 47.7% and 9.1% before 1993). However, these changes did not occur in all types of habitats. Significant changes were noted in the case of open drainage furrows (*p* < 0.001), streams (*p* < 0.001), road ditches (*p* < 0.01), spring heads (*p* < 0.01), and open drainage ditches (*p* < 0.01). No significant difference in the distributions of snail populations between the above two periods was noted for habitats located in pools and ponds, and in trampled areas.

**Table 5.** Distribution of 1987 habitats colonized by *Galba truncatula* on the 162 farms before 1993 and in 2013–2016 in relation to the type of habitat and the number of overwintering snails counted in March or April. The total number of snail habitats is given after each type of habitat (first column).

Parameters	Number of habitats colonized by *Galba truncatula*							
	Before 1993				From 2013 to 2016			
Snail density/m^2^	≤10	10.1–25	25.1–40	>40	≤10	10.1–25	25.1–40	>40
Type of snail habitat								
Drainage furrows (1598)	201	314	913	170	591	790	204	13
Road ditches (147)	86	61	0	0	94	43	10	0
Spring heads (102)	56	29	14	3	85	11	5	1
Streams (47)	7	23	10	7	11	29	7	0
Drainage ditches (41)	8	19	12	2	23	13	3	2
Pools and ponds (41)	37	4	0	0	38	2	1	0
Trampled areas (11)	11	0	0	0	7	4	0	0
All types of habitats (1987)	406	450	949	182	849	892	230	16
Frequency (%)	20.4	22.6	47.7	9.1	42.7	44.8	11.5	0.8

As for *G. truncatula*, a significant change (*p* < 0.001) in the overall distribution of *O. glabra* populations (Table 6) can be noted. Since 1993, the frequency of habitats containing fewer than 10 individuals/m^2^ has increased to 44.4% in 2013–2016 (versus 22.2% before 1993). At the same time, there was a decrease in the percentages of habitats containing 10.1 to 15 snails/m^2^ or a higher density (11.5% and 2.5%, respectively, in 2013–2016 compared to 22.1% and 10.2% before 1993). Significant changes were noted in the case of habitats located in open drainage furrows (*p* < 0.001), road ditches (*p* < 0.001), and streams (*p* < 0.01). In contrast, the other populations did not show any significant change in the distribution of their habitats over time when snail density was considered.

**Table 6.** Distribution of 866 habitats colonized by *Omphiscola glabra* on the 162 farms before 1993 and in 2013–2016 in relation to the type of habitat and the number of overwintering snails counted in March or April. The total number of snail habitats is given after each type of habitat (first column).

Parameters	Number of habitats colonized by *Omphiscola glabra*								
	Before 1993				From 2013 to 2016				
Snail density/m^2^	≤5	5.1–10	10.1–15	>15		≤5	5.1–10	10.1–15	>15
Type of snail habitat									
Drainage furrows (655)	101	304	167	83		261	289	86	19
Road ditches (77)	16	44	13	4		31	36	8	2
Drainage ditches (47)	35	11	1	0		40	7	0	0
Spring heads (34)	16	14	3	1		21	10	2	1
Streams (28)	4	16	7	1		12	13	3	0
Pools and ponds (23)	19	3	1	0		18	4	1	0
Trampled areas (2)	2	0	0	0		2	0	0	0
All types of habitats (866)	193	392	192	89		385	359	100	22
Frequency (%)	22.2	45.2	22.1	10.2		44.4	41.4	11.5	2.5

## Discussion

On the cristallophyllian and metamorphic soils of the Limousin region, both lymnaeid species showed a decline in the number of their populations over time. However, this decline rate presented variations according to snail species and the type of snail habitat (Table 7). Disappearance of numerous snail habitats from open drainage systems and road ditches was mainly due to mechanical cleaning of these sites, as this technique is increasingly applied in meadows and road ditches by herders and/or local administrative authorities [6, 21]. Even though use of mechanical cleaning once every two or three years seemed to have no effect on floristic richness of furrow and ditch banks [27], the removal of sludges by the backhoe and their transport out of cleaned furrows and ditches did not allow recolonization of these sites by surviving snails in most cases. Moreover, the period of ditch cleaning during summer drying and the absence of vegetation in these cleaned sites during this period had a negative effect on snail survival. Another cause of population decline was the progressive spreading of subsurface drainage systems in the meadows of 37 farms (out of 162 investigated) over time. The use of subsurface drains to manage excess soil water in these pastures led to total destruction of snail habitats generally located on the soil surface [7], including those located in and/or around spring heads on hillsides. The other four causes of population decline were of lesser importance and were more specific for each type of snail habitat. The creation of six ponds between 1993 and 2013–2016 in permanently waterlogged meadows led to the loss of 29 *G. truncatula* habitats and nine others colonized by *O. glabra*. Vegetation gyro-crushing applied once or twice a year in spring heads was responsible for destruction of numerous snail habitats, whatever the lymnaeid species. According to Rondelaud et al. [21], the destruction of rush beds around temporary springs has only become common since the 1990s. In 776 meadows studied by these authors on acidic soils, vegetation growing around 22.5% of temporary springs was gyro-crushed each year in 2008. The deposit of crushed plant fragments on spring heads and the surrounding zones had often led to snail disappearance because of the absence of unicellular algae that lymnaeids feed on [13, 25]. Water emptying from eight ponds, followed by their filling during the next 10–15 months had led to the destruction of 23 habitats previously colonized by either lymnaeid because of soil drying over a long period (>5 months). Lastly, the straightening of stream and pond banks by human or mechanical means was also responsible for destruction of snail habitats.

**Table 7.** Number of lost snail habitats in 2013–2016 in relation to the cause of population decline and the type of habitat. A/B, habitats colonized by *Galba truncatula*/sites inhabited by *Omphiscola glabra.*

Parameters	Number of lost habitats: A/B*					
	Open drainage		Road ditches (260/92)	Spring heads (192/83)	Streams (43/13)	Pools & ponds (30/9)
	Furrows (434/52)	Ditches (59/9)				
Mechanical cleaning	193/22	25/5	226/80	6/2	1/1	0/0
Subsurface drainage	166/13	21/2	0/0	11/9	1/0	0/0
Gyro-crushing of vegetation	43/6	4/1	27/11	172/66	4/1	3/2
Creation of ponds	21/7	5/1	0/0	¾	0/0	0/0
Drying out of ponds	0/0	0/0	0/0	0/0	0/0	19/4
Straightening of banks	0/0	3/0	0/0	0/0	38/11	7/3
Not determined	11/4	1/0	7/1	0/2	1/0	1/0

* Trampled areas: 4/3, subsurface drainage: 2/4, creation of ponds.

In the present study, the populations of *G. truncatula* were more affected by disappearance of their habitats than those of *O. glabra* (34.0% versus 23.4%, respectively: Table 2). This difference can partly be explained by the geographical location of snail habitats on acidic soils. As *G. truncatula* are more amphibious, its habitats are often confined to the peripheral extremity of open drainage furrows, while those colonized by *O. glabra* are located in the middle part of the same furrows [28, 29]. This location of habitats according to snail species was also noted in most road ditches and streams. In contrast, in pools and ponds, *G. truncatula* often colonize banks near water inlets, while the habitats of *O. glabra* are often located on banks or in zones with aquatic vegetation [31]. However, another explanation, suggesting a lower resistance of *G. truncatula* populations to unfavorable conditions, cannot be completely ruled out. In the field, *G. truncatula* did not resist competition from *O. glabra* in its habitats [15, 20] and the presence of both species in the same breeding boxes in the laboratory led to the rapid death of pre-adult or adult *G. truncatula* [5].

In *G. truncatula* populations reinvestigated in 2013–2016, overwintering snails were significantly less numerous in five types of habitats. This finding was also noted in the sites colonized by *O. glabra* but only in three types of habitats. These results are more difficult to comment. The current decrease in snail numbers might be due to the annual liming of acidic soils in permanent meadows, which was more scarcely applied in 2013–2016 than in the 1990s (D. Rondelaud, personal observation). However, two other, perhaps complementary, hypotheses may be proposed. First, this decrease in snail numbers might be due to an intense regeneration of vegetation around and/or in snail habitats after its mowing (vegetation was only mowed on acidic soils at the end of August or in September) and its development to more advanced stages during the next year under favorable climatic and hydrological conditions. As algal food production would be reduced or become null under this developing vegetation cover, this situation would lead to a limitation in snail numbers and even the disappearance of the population, like that reported by Moens [12] for *G. truncatula* populations in some Belgian meadows. Secondly, this decrease might also be due to variations in the frequency of iridovirosis in the case of *G. truncatula* (this virus was not still observed in *O. glabra*). As this viral disease was a common infection in the *G. truncatula* living in the Limousin region [6, 19], the regular occurrence of iridovirosis in these snail populations might be responsible for the decrease in snail numbers because most infected snails rapidly died over time [3].

Did the current decline in lymnaeid populations and the decrease in snail numbers observed in several types of habitats have an influence on local transmission of fasciolosis? This question is difficult to answer. The regular use of triclabendazole to treat ruminants against fasciolosis in the Limousin region has caused a gradual decrease in prevalence of this infection in cattle from the 2000s [18] and, consequently, a corresponding decrease in the number of snails naturally infected with *F. hepatica* (D. Rondelaud, personal observation). As mechanical cleaning in open drainage systems or the development of subsurface drainage was often limited to a single, or two or three swampy pastures in these farms, it was difficult to determine their impact on the prevalence of fasciolosis in these herds because most farmers used rotational grazing for their ruminants in relation to vegetation growth throughout the year. Further studies are still needed to determine whether disappearance of lymnaeid populations from meadows may have repercussions on the prevalence of fasciolosis in each cattle- or sheep-breeding farm.

In conclusion, both lymnaeid species in the Limousin region showed a decline in the number of their populations and also in the number of overwintering snails in numerous populations. Although these findings provide new data in the case of *G. truncatula*, they confirm, in contrast, the decline reported for *O. glabra* by Byrne et al. [4], Prié et al. [16], and Welter-Schultes [33] in other Western European countries. Verification of these preliminary results requires additional studies on the same lymnaeids but in other regions of France on acidic soils.
